# Bis[μ-4-(dimethyl­amino)­benzoato]-κ^3^ *O*,*O*′:*O*;κ^3^ *O*:*O*,*O*′-bis­{aqua­[4-(dimethyl­amino)­benzoato-κ^2^ *O*,*O*′](nicotinamide-κ*N* ^1^)cadmium(II)}

**DOI:** 10.1107/S160053681002163X

**Published:** 2010-06-16

**Authors:** Tuncer Hökelek, Yasemin Süzen, Barış Tercan, Özgür Aybirdi, Hacali Necefoğlu

**Affiliations:** a Department of Physics, Hacettepe University, 06800 Beytepe, Ankara, Turkey; b Department of Chemistry, Faculty of Science, Anadolu University, 26470 Yenibağlar, Eskişehir, Turkey; c Department of Physics, Karabük University, 78050, Karabük, Turkey; d Department of Chemistry, Kafkas University, 63100 Kars, Turkey

## Abstract

In the centrosymmetric dimeric Cd^II^ title compound, [Cd_2_(C_9_H_10_NO_2_)_4_(C_6_H_6_N_2_O)_2_(H_2_O)_2_], each seven-coordin­ated Cd^II^ atom is chelated by the carboxyl­ate groups of the two 4-(dimethyl­amino)­benzoate (DMAB) anions; the two monomeric units are bridged through the two O atoms of the two carboxyl groups. In the crystal structure, inter­molecular O—H⋯O, N—H⋯O and C—H⋯O hydrogen bonds link the mol­ecules into a three-dimensional network. π–π contacts between the pyridine rings [centroid–centroid distance = 3.974 (1) Å] may further stabilize the structure. Weak C—H⋯π inter­actions are also observed.

## Related literature

For the applications of transition metal complexes with mol­ecules in biological systems, see: Antolini *et al.* (1982 ▶). Benzoic acid derivatives such as 4-amino­benzoic acid are used extensively as bifunctional organic ligands in coordination chemistry due to the their various coordination modes, see: Amiraslanov *et al.* (1979 ▶); Chen & Chen (2002 ▶); Hauptmann *et al.* (2000 ▶). In pellagra disease, niacin deficiency leads to loss of copper from the body with high serum and urinary copper levels, see: Krishnamachari (1974 ▶). The nicotinic acid derivative *N*,*N*-diethyl­nicotinamide (DENA) is an important respiratory stimulant, see: Bigoli *et al.* (1972 ▶). For structure–function–coordination relationships of the aryl­carboxyl­ate ion in Mn^II^ complexes of benzoic acid derivatives, see: Adiwidjaja *et al.* (1978 ▶); Antsyshkina *et al.* (1980 ▶); Catterick *et al.* (1974 ▶); Shnulin *et al.* (1981 ▶). For related structures, see: Greenaway *et al.* (1984 ▶); Hökelek & Necefoğlu (1996 ▶); Hökelek *et al.* (2009*a* ▶,*b* ▶,*c* ▶,*d* ▶).
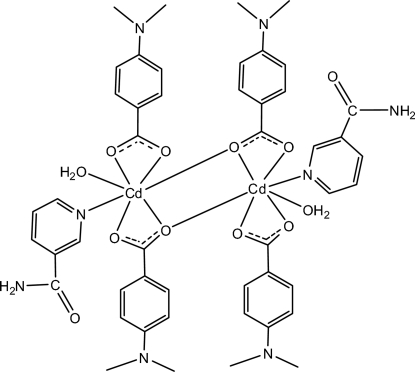


## Experimental

### 

#### Crystal data

[Cd_2_(C_9_H_10_NO_2_)_4_(C_6_H_6_N_2_O)_2_(H_2_O)_2_]
*M* *_r_* = 1161.83
Triclinic, 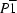

*a* = 9.5453 (2) Å
*b* = 10.2372 (2) Å
*c* = 13.5697 (3) Å
α = 74.102 (3)°
β = 79.479 (3)°
γ = 66.547 (2)°
*V* = 1165.85 (5) Å^3^
*Z* = 1
Mo *K*α radiation
μ = 0.99 mm^−1^
*T* = 100 K
0.36 × 0.24 × 0.13 mm

#### Data collection

Bruker Kappa APEXII CCD area-detector diffractometer
Absorption correction: multi-scan (*SADABS*; Bruker, 2005 ▶) *T*_min_ = 0.752, *T*_max_ = 0.879
21249 measured reflections
5862 independent reflections
5498 reflections with *I* > 2σ(*I*)
*R*_int_ = 0.026

#### Refinement

*R*[*F* ^2^ > 2σ(*F* ^2^)] = 0.022
*wR*(*F* ^2^) = 0.056
*S* = 1.06
5862 reflections
328 parameters
H atoms treated by a mixture of independent and constrained refinement
Δρ_max_ = 0.67 e Å^−3^
Δρ_min_ = −0.49 e Å^−3^

### 

Data collection: *APEX2* (Bruker, 2007 ▶); cell refinement: *SAINT* (Bruker, 2007 ▶); data reduction: *SAINT*; program(s) used to solve structure: *SHELXS97* (Sheldrick, 2008 ▶); program(s) used to refine structure: *SHELXL97* (Sheldrick, 2008 ▶); molecular graphics: *Mercury* (Macrae *et al.*, 2006 ▶); software used to prepare material for publication: *WinGX* (Farrugia, 1999 ▶) and *PLATON* (Spek, 2009 ▶).

## Supplementary Material

Crystal structure: contains datablocks I, global. DOI: 10.1107/S160053681002163X/xu2771sup1.cif

Structure factors: contains datablocks I. DOI: 10.1107/S160053681002163X/xu2771Isup2.hkl

Additional supplementary materials:  crystallographic information; 3D view; checkCIF report

## Figures and Tables

**Table 1:** Selected bond lengths (Å)

Cd1—O1	2.3511 (12)
Cd1—O2	2.3362 (12)
Cd1—O3	2.5705 (13)
Cd1—O4^i^	2.5762 (12)
Cd1—O6	2.3170 (12)
Cd1—N3	2.3339 (14)

Symmetry code: (i) 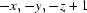.

**Table 2:** Hydrogen-bond geometry (Å, °) *Cg*2 and *Cg*3 are the centroids of the C11–C16 and N3/C19–C23 rings, respectively.

*D*—H⋯*A*	*D*—H	H⋯*A*	*D*⋯*A*	*D*—H⋯*A*
N4—H4*A*⋯O3^ii^	0.86	2.07	2.893 (2)	160
N4—H4*B*⋯O1^i^	0.86	2.24	2.993 (2)	147
O6—H61⋯O2^iii^	0.79 (3)	1.97 (3)	2.749 (2)	176 (2)
O6—H62⋯O5^iv^	0.82 (3)	1.91 (3)	2.703 (2)	163 (3)
C19—H19⋯O1^i^	0.93	2.44	3.302 (2)	155
C23—H23⋯O2^iii^	0.93	2.57	3.372 (2)	144
C9—H9*A*⋯*Cg*3^v^	0.96	2.61	3.434 (2)	144
C17—H17*B*⋯*Cg*2^vi^	0.96	2.98	3.887 (3)	159

Symmetry codes: (i) 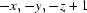; (ii) 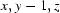; (iii) 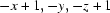; (iv) 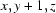; (v) 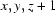; (vi) 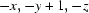.

**Fig. 1.**
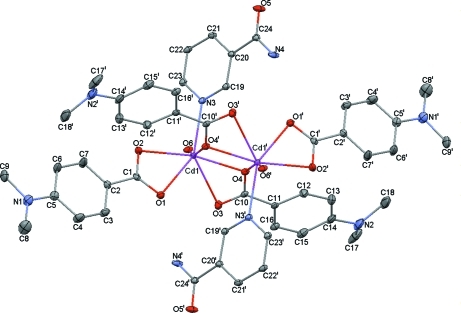
The molecular structure of the title molecule with the atom-numbering scheme. Displacement ellipsoids are drawn at the 40% probability level [symmetry code: (') -x, -y, 1 - z]. Hydrogen atoms are omitted for clarity.

## supplementary crystallographic information

### Comment

Transition metal complexes with biochemical molecules show interesting physical and/or chemical properties, through which they may find applications in biological systems (Antolini *et al.*, 1982). Some benzoic acid derivatives, such as 4-aminobenzoic acid, have been extensively reported in coordination chemistry, as bifunctional organic ligands, due to the varieties of their coordination modes (Chen & Chen, 2002; Amiraslanov *et al.*, 1979; Hauptmann *et al.*, 2000). Nicotinamide (NA) is one form of niacin. A deficiency of this vitamin leads to loss of copper from the body, known as pellagra disease. Victims of pellagra show unusually high serum and urinary copper levels (Krishnamachari, 1974). The nicotinic acid derivative *N*,*N*-Diethylnicotinamide (DENA) is an important respiratory stimulant (Bigoli *et al.*, 1972).

The structure-function-coordination relationships of the arylcarboxylate ion in Cd^II^ complexes of benzoic acid derivatives may also change depending on the nature and position of the substituted groups on the benzene ring, the nature of the additional ligand molecule or solvent, and the pH and temperature of synthesis as in Mn(II) complexes (Shnulin *et al.*, 1981; Antsyshkina *et al.*, 1980; Adiwidjaja *et al.*, 1978). When pyridine and its derivatives are used instead of water molecules, the structure is completely different (Catterick *et al.*, 1974). We report herein the synthesis and the structure of the title compound, (I).

The title compound, (I), consists of dimeric units located around a crystallographic symmetry centre and made up of two Cd cations, four 4-(dimethylamino)benzoato (DMAB) anions, two nicotinamide (NA) ligands and two water molecules (Fig. 1). Each Cd(II) unit is chelated by the carboxylate O atoms of the two DMAB anions, and the two monomeric units are bridged through the two oxygen atoms of the two carboxylate groups about an inversion center. The coordination number of each Cd^II^ atom is seven. The Cd1···Cd1^i^ distance is 3.8121 (2) Å and O4—Cd1—O4^i^ angle is 76.87 (4)° (symmetry code: (i) -*x*, -*y*, 1 - *z*).

The average Cd—O bond length (Table 1) is 2.4302 (12) Å, and the Cd atom is displaced out of the least-squares planes of the carboxylate groups (O1/C1/O2) and (O3/C10/O4) by 0.4160 (1) and 0.6395 (1) Å, respectively. In (I), the O1—Cd1—O2 and O3—Cd1—O4 angles are 55.96 (4) and 53.78 (4) °, respectively. The corresponding O—M—O (where *M* is a metal) angles are 52.91 (4)° and 53.96 (4)° in [Cd(C_8_H_5_O_3_)_2_(C_6_H_6_N_2_O)_2_(H_2_O)].H_2_O (Hökelek *et al.*, 2009*a*), 60.70 (4)° in [Co(C_9_H_10_NO_2_)_2_(C_6_H_6_N_2_O)(H_2_O)_2_] (Hökelek *et al.*, 2009*b*), 58.45 (9)° in [Mn(C_9_H_10_NO_2_)_2_(C_6_H_6_N_2_O)(H_2_O)_2_] (Hökelek *et al.*, 2009*c*), 60.03 (6)° in [Zn(C_8_H_8_NO_2_)_2_(C_6_H_6_N_2_O)_2_].H_2_O (Hökelek *et al.*, 2009 d), 58.3 (3)° in [Zn_2_(DENA)_2_(C_7_H_5_O_3_)_4_].2H_2_O (Hökelek & Necefoğlu, 1996) and 55.2 (1)° in [Cu(Asp)_2_(py)_2_] (where Asp is acetylsalicylate and py is pyridine) (Greenaway *et al.*, 1984).

The dihedral angles between the planar carboxylate groups and the adjacent benzene rings A (C2—C7) and B (C11—C16) are 11.48 (17) and 12.78 (13) °, respectively, while those between rings A, B, C (N3/C19—C23), D (Cd1/O1/O2/C1) and E (Cd1/O3/O4/C10) are A/B = 78.35 (7), A/C = 68.85 (6), B/C = 75.32 (6) and D/E = 61.98 (5)°.

In the crystal structure, intermolecular O—H···O, N—H···O and C—H···O hydrogen bonds (Table 2) link the molecules into a three dimensional network, in which they may be effective in the stabilization of the structure. The π–π contact between the pyridine rings, *Cg*3—*Cg*3^i^ [symmetry code: (i) 1 - *x*, -1 - *y*, 1 - *z*, where *Cg*3 is the centroid of the ring C (N3/C19—C23)] may further stabilize the structure, with centroid-centroid distance of 3.974 (1) Å. There also exist two weak C—H···π interactions (Table 2).

### Experimental

The title compound was prepared by the reaction of 3CdSO_4_.H_2_O (1.28 g, 5 mmol) in H_2_O (30 ml) and NA (1.22 g, 10 mmol) in H_2_O (20 ml) with sodium 4-(dimethylamino)benzoate (1.88 g, 10 mmol) in H_2_O (150 ml). The mixture was filtered and set aside to crystallize at ambient temperature for one week, giving colorless single crystals.

### Refinement

Atoms H61 and H62 were located in a difference Fourier map and refined isotropically. The remaining H atoms were positioned geometrically with N—H = 0.86 Å (for NH_2_) and C—H = 0.93 and 0.96 Å for aromatic and methyl H atoms, respectively, and constrained to ride on their parent atoms, with *U*_iso_(H) = *xU*_eq_(C,N), where *x* = 1.5 for methyl H and *x* = 1.2 for all other H atoms.

### Crystal data

**Table:**

[Cd_2_(C_9_H_10_NO_2_)_4_(C_6_H_6_N_2_O)_2_(H_2_O)_2_]	*Z* = 1
*M**_r_* = 1161.83	*F*(000) = 592
Triclinic, *P*1	*D*_x_ = 1.655 Mg m^−^^3^
Hall symbol: -P 1	Mo *K*α radiation, λ = 0.71073 Å
*a* = 9.5453 (2) Å	Cell parameters from 9880 reflections
*b* = 10.2372 (2) Å	θ = 2.4–28.5°
*c* = 13.5697 (3) Å	µ = 0.99 mm^−^^1^
α = 74.102 (3)°	*T* = 100 K
β = 79.479 (3)°	Block, colorless
γ = 66.547 (2)°	0.36 × 0.24 × 0.13 mm
*V* = 1165.85 (5) Å^3^	

### Data collection

**Table:**

Bruker Kappa APEXII CCD area-detector diffractometer	5862 independent reflections
Radiation source: fine-focus sealed tube	5498 reflections with *I* > 2σ(*I*)
graphite	*R*_int_ = 0.026
φ and ω scans	θ_max_ = 28.5°, θ_min_ = 1.6°
Absorption correction: multi-scan (*SADABS*; Bruker, 2005)	*h* = −12→12
*T*_min_ = 0.752, *T*_max_ = 0.879	*k* = −13→13
21249 measured reflections	*l* = −18→18

### Refinement

**Table:**

Refinement on *F*^2^	Primary atom site location: structure-invariant direct methods
Least-squares matrix: full	Secondary atom site location: difference Fourier map
*R*[*F*^2^ > 2σ(*F*^2^)] = 0.022	Hydrogen site location: inferred from neighbouring sites
*wR*(*F*^2^) = 0.056	H atoms treated by a mixture of independent and constrained refinement
*S* = 1.06	*w* = 1/[σ^2^(*F*_o_^2^) + (0.0271*P*)^2^ + 0.7237*P*] where *P* = (*F*_o_^2^ + 2*F*_c_^2^)/3
5862 reflections	(Δ/σ)_max_ < 0.001
328 parameters	Δρ_max_ = 0.67 e Å^−^^3^
0 restraints	Δρ_min_ = −0.49 e Å^−^^3^

### Special details

**Table:**

Geometry. All e.s.d.'s (except the e.s.d. in the dihedral angle between two l.s. planes) are estimated using the full covariance matrix. The cell e.s.d.'s are taken into account individually in the estimation of e.s.d.'s in distances, angles and torsion angles; correlations between e.s.d.'s in cell parameters are only used when they are defined by crystal symmetry. An approximate (isotropic) treatment of cell e.s.d.'s is used for estimating e.s.d.'s involving l.s. planes.
Refinement. Refinement of *F*^2^ against ALL reflections. The weighted *R*-factor *wR* and goodness of fit *S* are based on *F*^2^, conventional *R*-factors *R* are based on *F*, with *F* set to zero for negative *F*^2^. The threshold expression of *F*^2^ > σ(*F*^2^) is used only for calculating *R*-factors(gt) *etc*. and is not relevant to the choice of reflections for refinement. *R*-factors based on *F*^2^ are statistically about twice as large as those based on *F*, and *R*- factors based on ALL data will be even larger.

### Fractional atomic coordinates and isotropic or equivalent isotropic displacement parameters (Å^2^)

**Table:**

	*x*	*y*	*z*	*U*_iso_*/*U*_eq_	
Cd1	0.180220 (12)	0.040917 (12)	0.492667 (9)	0.01404 (4)	
O1	0.10108 (14)	0.14484 (13)	0.63748 (9)	0.0207 (2)	
O2	0.33823 (14)	−0.01006 (15)	0.62185 (10)	0.0221 (3)	
O3	0.02478 (14)	0.30555 (14)	0.40644 (10)	0.0212 (2)	
O4	−0.02665 (13)	0.11559 (13)	0.40059 (9)	0.0189 (2)	
O5	0.35463 (15)	−0.60232 (14)	0.34334 (13)	0.0320 (3)	
O6	0.34839 (15)	0.12770 (14)	0.37393 (10)	0.0208 (3)	
H61	0.438 (3)	0.091 (3)	0.374 (2)	0.039 (7)*	
H62	0.331 (3)	0.215 (3)	0.364 (2)	0.042 (7)*	
N1	0.2767 (2)	−0.0371 (2)	1.10325 (13)	0.0338 (4)	
N2	−0.2931 (2)	0.5498 (2)	−0.01791 (15)	0.0481 (6)	
N3	0.32213 (15)	−0.17835 (15)	0.44145 (11)	0.0161 (3)	
N4	0.12133 (18)	−0.45388 (19)	0.38859 (17)	0.0367 (5)	
H4A	0.0796	−0.5110	0.3814	0.044*	
H4B	0.0655	−0.3738	0.4075	0.044*	
C1	0.22674 (19)	0.06515 (18)	0.67584 (13)	0.0172 (3)	
C2	0.24275 (19)	0.04966 (19)	0.78525 (13)	0.0188 (3)	
C3	0.1236 (2)	0.1251 (2)	0.84793 (15)	0.0297 (4)	
H3	0.0358	0.1959	0.8188	0.036*	
C4	0.1318 (2)	0.0978 (3)	0.95279 (15)	0.0358 (5)	
H4	0.0498	0.1506	0.9925	0.043*	
C5	0.2614 (2)	−0.0079 (2)	1.00013 (14)	0.0241 (4)	
C6	0.3803 (2)	−0.0844 (3)	0.93693 (17)	0.0432 (6)	
H6	0.4680	−0.1559	0.9658	0.052*	
C7	0.3707 (2)	−0.0565 (3)	0.83278 (17)	0.0414 (6)	
H7	0.4520	−0.1101	0.7930	0.050*	
C8	0.1518 (3)	0.0277 (3)	1.17494 (18)	0.0522 (7)	
H8A	0.1926	0.0409	1.2296	0.078*	
H8B	0.0955	−0.0355	1.2029	0.078*	
H8C	0.0849	0.1207	1.1397	0.078*	
C9	0.4091 (3)	−0.1524 (3)	1.14836 (17)	0.0412 (5)	
H9A	0.4063	−0.1499	1.2189	0.062*	
H9B	0.5002	−0.1395	1.1114	0.062*	
H9C	0.4092	−0.2449	1.1449	0.062*	
C10	−0.04015 (17)	0.25041 (18)	0.36627 (13)	0.0164 (3)	
C11	−0.12533 (19)	0.33661 (18)	0.27396 (13)	0.0188 (3)	
C12	−0.1946 (2)	0.2771 (2)	0.22633 (16)	0.0303 (4)	
H12	−0.2022	0.1873	0.2590	0.036*	
C13	−0.2528 (3)	0.3473 (3)	0.13181 (18)	0.0400 (5)	
H13	−0.2986	0.3040	0.1022	0.048*	
C14	−0.2439 (2)	0.4826 (3)	0.07997 (16)	0.0353 (5)	
C15	−0.1832 (2)	0.5474 (2)	0.13103 (16)	0.0316 (4)	
H15	−0.1824	0.6404	0.1010	0.038*	
C16	−0.1245 (2)	0.4756 (2)	0.22524 (14)	0.0231 (4)	
H16	−0.0835	0.5207	0.2569	0.028*	
C17	−0.2400 (3)	0.6651 (4)	−0.07824 (19)	0.0612 (9)	
H17A	−0.2756	0.6970	−0.1457	0.092*	
H17B	−0.1300	0.6285	−0.0838	0.092*	
H17C	−0.2792	0.7458	−0.0451	0.092*	
C18	−0.3162 (4)	0.4603 (4)	−0.0761 (2)	0.0700 (11)	
H18A	−0.3484	0.5187	−0.1424	0.105*	
H18B	−0.3935	0.4231	−0.0396	0.105*	
H18C	−0.2219	0.3802	−0.0846	0.105*	
C19	0.25695 (18)	−0.26001 (17)	0.41870 (12)	0.0158 (3)	
H19	0.1506	−0.2288	0.4251	0.019*	
C20	0.34143 (18)	−0.38911 (17)	0.38596 (13)	0.0160 (3)	
C21	0.50027 (19)	−0.43196 (18)	0.37210 (14)	0.0192 (3)	
H21	0.5599	−0.5158	0.3479	0.023*	
C22	0.56831 (19)	−0.34787 (18)	0.39492 (14)	0.0200 (3)	
H22	0.6742	−0.3742	0.3861	0.024*	
C23	0.47585 (18)	−0.22380 (18)	0.43116 (13)	0.0183 (3)	
H23	0.5221	−0.1699	0.4491	0.022*	
C24	0.27089 (19)	−0.48910 (18)	0.37050 (13)	0.0180 (3)	

### Atomic displacement parameters (Å^2^)

**Table:**

	*U*^11^	*U*^22^	*U*^33^	*U*^12^	*U*^13^	*U*^23^
Cd1	0.00975 (6)	0.01423 (6)	0.01936 (7)	−0.00424 (4)	−0.00041 (4)	−0.00645 (4)
O1	0.0180 (6)	0.0199 (6)	0.0216 (6)	−0.0039 (5)	−0.0013 (5)	−0.0055 (5)
O2	0.0146 (6)	0.0317 (7)	0.0235 (6)	−0.0076 (5)	0.0011 (5)	−0.0147 (5)
O3	0.0175 (6)	0.0234 (6)	0.0251 (6)	−0.0106 (5)	−0.0027 (5)	−0.0038 (5)
O4	0.0146 (5)	0.0156 (5)	0.0242 (6)	−0.0040 (4)	−0.0016 (5)	−0.0032 (5)
O5	0.0226 (7)	0.0207 (6)	0.0575 (10)	−0.0107 (5)	0.0111 (6)	−0.0216 (6)
O6	0.0140 (6)	0.0149 (6)	0.0319 (7)	−0.0055 (5)	0.0024 (5)	−0.0054 (5)
N1	0.0317 (9)	0.0417 (10)	0.0189 (8)	−0.0081 (8)	0.0012 (7)	−0.0031 (7)
N2	0.0338 (10)	0.0590 (14)	0.0280 (10)	0.0090 (9)	−0.0135 (8)	−0.0055 (9)
N3	0.0130 (6)	0.0142 (6)	0.0208 (7)	−0.0052 (5)	0.0000 (5)	−0.0043 (5)
N4	0.0140 (7)	0.0274 (8)	0.0800 (14)	−0.0064 (6)	0.0000 (8)	−0.0343 (9)
C1	0.0168 (7)	0.0183 (7)	0.0206 (8)	−0.0100 (6)	0.0016 (6)	−0.0075 (6)
C2	0.0173 (8)	0.0225 (8)	0.0199 (8)	−0.0098 (7)	0.0013 (6)	−0.0077 (6)
C3	0.0241 (9)	0.0290 (10)	0.0219 (9)	0.0031 (8)	0.0010 (7)	−0.0050 (7)
C4	0.0273 (10)	0.0416 (12)	0.0205 (9)	0.0025 (9)	0.0055 (8)	−0.0067 (8)
C5	0.0227 (9)	0.0290 (9)	0.0204 (8)	−0.0116 (7)	0.0015 (7)	−0.0040 (7)
C6	0.0231 (10)	0.0654 (16)	0.0308 (11)	0.0066 (10)	−0.0103 (8)	−0.0243 (11)
C7	0.0182 (9)	0.0676 (16)	0.0304 (11)	0.0054 (10)	−0.0056 (8)	−0.0284 (11)
C8	0.0413 (14)	0.0785 (19)	0.0211 (10)	−0.0141 (13)	0.0112 (9)	−0.0075 (11)
C9	0.0452 (13)	0.0485 (13)	0.0272 (10)	−0.0092 (11)	−0.0155 (9)	−0.0083 (10)
C10	0.0094 (7)	0.0177 (7)	0.0194 (8)	−0.0030 (6)	0.0010 (6)	−0.0045 (6)
C11	0.0132 (7)	0.0172 (7)	0.0217 (8)	0.0000 (6)	−0.0023 (6)	−0.0054 (6)
C12	0.0327 (10)	0.0223 (9)	0.0367 (11)	−0.0064 (8)	−0.0141 (8)	−0.0060 (8)
C13	0.0404 (12)	0.0380 (12)	0.0417 (12)	−0.0030 (10)	−0.0223 (10)	−0.0141 (10)
C14	0.0227 (9)	0.0399 (11)	0.0263 (10)	0.0078 (8)	−0.0082 (8)	−0.0061 (8)
C15	0.0222 (9)	0.0298 (10)	0.0290 (10)	−0.0014 (8)	−0.0015 (8)	0.0023 (8)
C16	0.0169 (8)	0.0226 (8)	0.0262 (9)	−0.0050 (7)	−0.0004 (7)	−0.0039 (7)
C17	0.0276 (12)	0.083 (2)	0.0278 (12)	0.0100 (12)	−0.0016 (9)	0.0126 (12)
C18	0.0556 (17)	0.085 (2)	0.0421 (15)	0.0227 (16)	−0.0289 (13)	−0.0291 (15)
C19	0.0124 (7)	0.0151 (7)	0.0199 (8)	−0.0058 (6)	0.0002 (6)	−0.0040 (6)
C20	0.0143 (7)	0.0138 (7)	0.0199 (8)	−0.0053 (6)	−0.0002 (6)	−0.0043 (6)
C21	0.0136 (7)	0.0155 (7)	0.0257 (8)	−0.0032 (6)	0.0017 (6)	−0.0058 (6)
C22	0.0106 (7)	0.0176 (8)	0.0293 (9)	−0.0042 (6)	0.0002 (6)	−0.0038 (7)
C23	0.0139 (7)	0.0169 (7)	0.0248 (8)	−0.0072 (6)	−0.0011 (6)	−0.0038 (6)
C24	0.0164 (7)	0.0141 (7)	0.0236 (8)	−0.0057 (6)	−0.0010 (6)	−0.0045 (6)

### Geometric parameters (Å, °)

**Table:**

Cd1—O1	2.3511 (12)	C8—H8A	0.9600
Cd1—O2	2.3362 (12)	C8—H8B	0.9600
Cd1—O3	2.5705 (13)	C8—H8C	0.9600
Cd1—O4^i^	2.5762 (12)	C9—H9A	0.9600
Cd1—O6	2.3170 (12)	C9—H9B	0.9600
Cd1—N3	2.3339 (14)	C9—H9C	0.9600
Cd1—C1	2.6955 (17)	C11—C10	1.485 (2)
O1—C1	1.262 (2)	C11—C12	1.385 (3)
O2—C1	1.278 (2)	C11—C16	1.398 (2)
O3—C10	1.253 (2)	C12—C13	1.381 (3)
O4—Cd1	2.2849 (12)	C12—H12	0.9300
O4—Cd1^i^	2.5762 (12)	C13—C14	1.400 (3)
O4—C10	1.291 (2)	C13—H13	0.9300
O5—C24	1.228 (2)	C14—N2	1.388 (3)
O6—H61	0.78 (3)	C15—C14	1.400 (3)
O6—H62	0.81 (3)	C15—H15	0.9300
N1—C5	1.371 (2)	C16—C15	1.381 (3)
N1—C8	1.452 (3)	C16—H16	0.9300
N1—C9	1.437 (3)	C17—N2	1.455 (4)
N3—C19	1.344 (2)	C17—H17A	0.9600
N3—C23	1.344 (2)	C17—H17B	0.9600
N4—C24	1.319 (2)	C17—H17C	0.9600
N4—H4A	0.8600	C18—N2	1.460 (4)
N4—H4B	0.8600	C18—H18A	0.9600
C1—C2	1.479 (2)	C18—H18B	0.9600
C2—C3	1.386 (2)	C18—H18C	0.9600
C2—C7	1.391 (3)	C19—C20	1.392 (2)
C3—C4	1.384 (3)	C19—H19	0.9300
C3—H3	0.9300	C20—C21	1.392 (2)
C4—H4	0.9300	C20—C24	1.504 (2)
C5—C4	1.399 (3)	C21—C22	1.387 (2)
C5—C6	1.394 (3)	C21—H21	0.9300
C6—C7	1.376 (3)	C22—H22	0.9300
C6—H6	0.9300	C23—C22	1.388 (2)
C7—H7	0.9300	C23—H23	0.9300
			
O1—Cd1—O3	80.40 (4)	C7—C6—C5	121.4 (2)
O1—Cd1—O4^i^	81.03 (4)	C7—C6—H6	119.3
O1—Cd1—C1	27.90 (5)	C2—C7—H7	119.1
O2—Cd1—O1	55.96 (4)	C6—C7—C2	121.85 (19)
O2—Cd1—O3	121.05 (4)	C6—C7—H7	119.1
O2—Cd1—O4^i^	95.15 (4)	N1—C8—H8A	109.5
O2—Cd1—C1	28.30 (5)	N1—C8—H8B	109.5
O3—Cd1—O4^i^	116.80 (4)	N1—C8—H8C	109.5
O3—Cd1—C1	103.06 (5)	H8A—C8—H8B	109.5
O4—Cd1—O1	108.51 (4)	H8A—C8—H8C	109.5
O4—Cd1—O2	163.91 (4)	H8B—C8—H8C	109.5
O4—Cd1—O3	53.78 (4)	N1—C9—H9A	109.5
O4—Cd1—O4^i^	76.87 (4)	N1—C9—H9B	109.5
O4—Cd1—O6	102.15 (5)	N1—C9—H9C	109.5
O4—Cd1—N3	98.43 (5)	H9A—C9—H9B	109.5
O4—Cd1—C1	135.79 (5)	H9A—C9—H9C	109.5
O4^i^—Cd1—C1	85.18 (4)	H9B—C9—H9C	109.5
O6—Cd1—O1	113.98 (5)	O3—C10—O4	120.68 (15)
O6—Cd1—O2	89.36 (5)	O3—C10—C11	120.28 (15)
O6—Cd1—O3	73.12 (4)	O4—C10—C11	118.91 (15)
O6—Cd1—O4^i^	163.97 (4)	C12—C11—C10	121.50 (16)
O6—Cd1—N3	84.01 (5)	C12—C11—C16	117.17 (17)
O6—Cd1—C1	105.44 (5)	C16—C11—C10	120.97 (16)
N3—Cd1—O1	142.57 (5)	C11—C12—H12	119.0
N3—Cd1—O2	93.88 (5)	C13—C12—C11	121.9 (2)
N3—Cd1—O3	137.02 (4)	C13—C12—H12	119.0
N3—Cd1—O4^i^	80.34 (4)	C12—C13—C14	121.0 (2)
N3—Cd1—C1	118.10 (5)	C12—C13—H13	119.5
C1—O1—Cd1	91.41 (10)	C14—C13—H13	119.5
C1—O2—Cd1	91.67 (10)	N2—C14—C13	121.5 (2)
C10—O3—Cd1	85.54 (10)	N2—C14—C15	121.4 (2)
Cd1—O4—Cd1^i^	103.13 (4)	C13—C14—C15	117.13 (18)
C10—O4—Cd1	97.69 (10)	C14—C15—H15	119.4
C10—O4—Cd1^i^	140.80 (10)	C16—C15—C14	121.2 (2)
Cd1—O6—H61	124 (2)	C16—C15—H15	119.4
Cd1—O6—H62	116.2 (19)	C11—C16—H16	119.3
H61—O6—H62	104 (3)	C15—C16—C11	121.40 (19)
C5—N1—C9	120.90 (18)	C15—C16—H16	119.3
C5—N1—C8	122.36 (19)	N2—C17—H17A	109.5
C9—N1—C8	115.94 (18)	N2—C17—H17B	109.5
C14—N2—C17	117.8 (2)	N2—C17—H17C	109.5
C14—N2—C18	117.6 (2)	H17A—C17—H17B	109.5
C17—N2—C18	115.8 (2)	H17A—C17—H17C	109.5
C19—N3—Cd1	122.92 (10)	H17B—C17—H17C	109.5
C23—N3—Cd1	119.01 (11)	N2—C18—H18A	109.5
C23—N3—C19	118.04 (14)	N2—C18—H18B	109.5
C24—N4—H4A	120.0	N2—C18—H18C	109.5
C24—N4—H4B	120.0	H18A—C18—H18B	109.5
H4A—N4—H4B	120.0	H18A—C18—H18C	109.5
O1—C1—Cd1	60.69 (9)	H18B—C18—H18C	109.5
O1—C1—O2	119.93 (15)	N3—C19—C20	122.96 (14)
O1—C1—C2	120.45 (15)	N3—C19—H19	118.5
O2—C1—Cd1	60.04 (9)	C20—C19—H19	118.5
O2—C1—C2	119.48 (15)	C19—C20—C21	118.29 (15)
C2—C1—Cd1	167.27 (11)	C19—C20—C24	123.42 (14)
C3—C2—C1	121.73 (16)	C21—C20—C24	118.20 (14)
C3—C2—C7	116.93 (17)	C20—C21—H21	120.5
C7—C2—C1	120.76 (16)	C22—C21—C20	119.03 (15)
C2—C3—H3	119.1	C22—C21—H21	120.5
C4—C3—C2	121.74 (18)	C21—C22—C23	118.92 (15)
C4—C3—H3	119.1	C21—C22—H22	120.5
C3—C4—C5	121.17 (18)	C23—C22—H22	120.5
C3—C4—H4	119.4	N3—C23—C22	122.66 (15)
C5—C4—H4	119.4	N3—C23—H23	118.7
N1—C5—C4	123.52 (18)	C22—C23—H23	118.7
N1—C5—C6	119.59 (18)	O5—C24—N4	122.03 (16)
C6—C5—C4	116.89 (18)	O5—C24—C20	118.99 (15)
C5—C6—H6	119.3	N4—C24—C20	118.97 (15)
			
O2—Cd1—O1—C1	−5.81 (9)	Cd1^i^—O4—Cd1—C1	−68.68 (7)
O3—Cd1—O1—C1	−144.01 (10)	C10—O4—Cd1—O1	70.86 (10)
O4—Cd1—O1—C1	169.50 (9)	C10—O4—Cd1—O2	85.00 (18)
O4^i^—Cd1—O1—C1	96.68 (10)	C10—O4—Cd1—O3	8.44 (9)
O6—Cd1—O1—C1	−77.44 (10)	C10—O4—Cd1—O4^i^	146.56 (11)
N3—Cd1—O1—C1	35.73 (13)	C10—O4—Cd1—O6	−49.84 (10)
O1—Cd1—O2—C1	5.74 (9)	C10—O4—Cd1—N3	−135.48 (10)
O3—Cd1—O2—C1	55.83 (11)	C10—O4—Cd1—C1	77.88 (11)
O4—Cd1—O2—C1	−10.5 (2)	Cd1—O4—C10—O3	−16.40 (16)
O4^i^—Cd1—O2—C1	−69.80 (10)	Cd1^i^—O4—C10—O3	105.49 (18)
O6—Cd1—O2—C1	125.61 (10)	Cd1—O4—C10—C11	159.57 (12)
N3—Cd1—O2—C1	−150.44 (10)	Cd1^i^—O4—C10—C11	−78.5 (2)
O1—Cd1—O3—C10	−130.16 (10)	C8—N1—C5—C4	7.3 (4)
O2—Cd1—O3—C10	−170.30 (9)	C8—N1—C5—C6	−173.6 (3)
O4—Cd1—O3—C10	−8.64 (9)	C9—N1—C5—C4	176.6 (2)
O4^i^—Cd1—O3—C10	−55.38 (10)	C9—N1—C5—C6	−4.3 (3)
O6—Cd1—O3—C10	111.01 (10)	Cd1—N3—C19—C20	178.53 (12)
N3—Cd1—O3—C10	50.07 (11)	C23—N3—C19—C20	0.5 (2)
C1—Cd1—O3—C10	−146.56 (9)	Cd1—N3—C23—C22	−175.85 (13)
O1—Cd1—N3—C19	104.17 (13)	C19—N3—C23—C22	2.3 (2)
O1—Cd1—N3—C23	−77.81 (14)	Cd1—C1—C2—C3	−93.7 (6)
O2—Cd1—N3—C19	137.60 (13)	Cd1—C1—C2—C7	77.3 (6)
O2—Cd1—N3—C23	−44.39 (13)	O1—C1—C2—C3	−2.3 (3)
O3—Cd1—N3—C19	−76.20 (14)	O1—C1—C2—C7	168.71 (19)
O3—Cd1—N3—C23	101.82 (13)	O2—C1—C2—C3	−177.86 (17)
O4—Cd1—N3—C19	−32.01 (13)	O2—C1—C2—C7	−6.9 (3)
O4^i^—Cd1—N3—C19	43.03 (12)	C1—C2—C3—C4	172.1 (2)
O4—Cd1—N3—C23	146.00 (12)	C7—C2—C3—C4	0.8 (3)
O4^i^—Cd1—N3—C23	−138.96 (13)	C1—C2—C7—C6	−172.4 (2)
O6—Cd1—N3—C19	−133.45 (13)	C3—C2—C7—C6	−1.0 (4)
O6—Cd1—N3—C23	44.56 (12)	C2—C3—C4—C5	−0.1 (4)
C1—Cd1—N3—C19	122.22 (12)	N1—C5—C4—C3	178.6 (2)
C1—Cd1—N3—C23	−59.76 (13)	C6—C5—C4—C3	−0.5 (4)
O1—Cd1—C1—O2	−169.81 (16)	N1—C5—C6—C7	−178.8 (3)
O1—Cd1—C1—C2	98.7 (5)	C4—C5—C6—C7	0.4 (4)
O2—Cd1—C1—O1	169.81 (16)	C5—C6—C7—C2	0.4 (4)
O2—Cd1—C1—C2	−91.5 (5)	C12—C11—C10—O3	178.21 (17)
O3—Cd1—C1—O1	36.50 (10)	C12—C11—C10—O4	2.2 (2)
O3—Cd1—C1—O2	−133.31 (10)	C16—C11—C10—O3	5.3 (2)
O3—Cd1—C1—C2	135.2 (5)	C16—C11—C10—O4	−170.72 (15)
O4—Cd1—C1—O1	−14.35 (13)	C10—C11—C12—C13	−169.52 (19)
O4^i^—Cd1—C1—O1	−79.92 (9)	C16—C11—C12—C13	3.7 (3)
O4—Cd1—C1—O2	175.84 (9)	C10—C11—C16—C15	170.03 (16)
O4^i^—Cd1—C1—O2	110.28 (10)	C12—C11—C16—C15	−3.2 (3)
O4—Cd1—C1—C2	84.4 (5)	C11—C12—C13—C14	0.0 (3)
O4^i^—Cd1—C1—C2	18.8 (5)	C12—C13—C14—N2	175.8 (2)
O6—Cd1—C1—O1	112.31 (10)	C12—C13—C14—C15	−4.1 (3)
O6—Cd1—C1—O2	−57.50 (10)	C13—C14—N2—C17	−162.1 (2)
O6—Cd1—C1—C2	−149.0 (5)	C13—C14—N2—C18	−15.8 (3)
N3—Cd1—C1—O1	−156.28 (9)	C15—C14—N2—C17	17.8 (3)
N3—Cd1—C1—O2	33.92 (11)	C15—C14—N2—C18	164.1 (2)
N3—Cd1—C1—C2	−57.5 (5)	C16—C15—C14—N2	−175.34 (19)
Cd1—O1—C1—O2	10.19 (16)	C16—C15—C14—C13	4.6 (3)
Cd1—O1—C1—C2	−165.37 (13)	C11—C16—C15—C14	−1.0 (3)
Cd1—O2—C1—O1	−10.26 (16)	N3—C19—C20—C21	−2.8 (2)
Cd1—O2—C1—C2	165.34 (13)	N3—C19—C20—C24	173.63 (15)
Cd1—O3—C10—O4	14.44 (14)	C19—C20—C21—C22	2.3 (2)
Cd1—O3—C10—C11	−161.46 (14)	C24—C20—C21—C22	−174.27 (16)
Cd1^i^—O4—Cd1—O1	−75.71 (5)	C19—C20—C24—O5	−178.59 (17)
Cd1^i^—O4—Cd1—O2	−61.56 (17)	C19—C20—C24—N4	0.0 (3)
Cd1^i^—O4—Cd1—O3	−138.13 (6)	C21—C20—C24—O5	−2.2 (3)
Cd1^i^—O4—Cd1—O4^i^	0.0	C21—C20—C24—N4	176.46 (18)
Cd1^i^—O4—Cd1—O6	163.60 (4)	C20—C21—C22—C23	0.2 (3)
Cd1^i^—O4—Cd1—N3	77.95 (5)	N3—C23—C22—C21	−2.6 (3)

Symmetry codes: (i) −*x*, −*y*, −*z*+1.

### Hydrogen-bond geometry (Å, °)

**Table:**

Cg2 and Cg3 are the centroids of the C11–C16 and N3/C19–C23 rings, respectively.

**Table:**

*D*—H···*A*	*D*—H	H···*A*	*D*···*A*	*D*—H···*A*
N4—H4A···O3^ii^	0.86	2.07	2.893 (2)	160
N4—H4B···O1^i^	0.86	2.24	2.993 (2)	147
O6—H61···O2^iii^	0.79 (3)	1.97 (3)	2.749 (2)	176 (2)
O6—H62···O5^iv^	0.82 (3)	1.91 (3)	2.703 (2)	163 (3)
C19—H19···O1^i^	0.93	2.44	3.302 (2)	155
C23—H23···O2^iii^	0.93	2.57	3.372 (2)	144
C9—H9A···Cg3^v^	0.96	2.61	3.434 (2)	144
C17—H17B···Cg2^vi^	0.96	2.98	3.887 (3)	159

Symmetry codes: (ii) *x*, *y*−1, *z*; (i) −*x*, −*y*, −*z*+1; (iii) −*x*+1, −*y*, −*z*+1; (iv) *x*, *y*+1, *z*; (v) *x*, *y*, *z*+1; (vi) −*x*, −*y*+1, −*z*.

## References

[bb1] Adiwidjaja, G., Rossmanith, E. & Küppers, H. (1978). *Acta Cryst.* B**34**, 3079–3083.

[bb2] Amiraslanov, I. R., Mamedov, Kh. S., Movsumov, E. M., Musaev, F. N. & Nadzhafov, G. N. (1979). *Zh. Strukt. Khim.* **20**, 1075–1080.

[bb3] Antolini, L., Battaglia, L. P., Corradi, A. B., Marcotrigiano, G., Menabue, L., Pellacani, G. C. & Saladini, M. (1982). *Inorg. Chem.* **21**, 1391–1395.

[bb4] Antsyshkina, A. S., Chiragov, F. M. & Poray-Koshits, M. A. (1980). *Koord. Khim.* **15**, 1098–1103.

[bb5] Bigoli, F., Braibanti, A., Pellinghelli, M. A. & Tiripicchio, A. (1972). *Acta Cryst.* B**28**, 962–966.

[bb6] Bruker (2005). *SADABS* Bruker AXS Inc. Madison, Wisconsin, USA.

[bb7] Bruker (2007). *APEX2* and *SAINT* Bruker AXS Inc., Madison, Wisconsin, USA.

[bb8] Catterick, J., Hursthouse, M. B., New, D. B. & Thorhton, P. (1974). *J. Chem. Soc. Chem. Commun.* pp. 843–844.

[bb9] Chen, H. J. & Chen, X. M. (2002). *Inorg. Chim. Acta*, **329**, 13–21.

[bb10] Farrugia, L. J. (1999). *J. Appl. Cryst.* **32**, 837–838.

[bb11] Greenaway, F. T., Pezeshk, A., Cordes, A. W., Noble, M. C. & Sorenson, J. R. J. (1984). *Inorg. Chim. Acta*, **93**, 67–71.

[bb12] Hauptmann, R., Kondo, M. & Kitagawa, S. (2000). *Z. Kristallogr. New Cryst. Struct.* **215**, 169–172.

[bb13] Hökelek, T., Dal, H., Tercan, B., Aybirdi, Ö. & Necefoğlu, H. (2009*b*). *Acta Cryst.* E**65**, m627–m628. 10.1107/S1600536809016602 PMC2969611 21582996

[bb14] Hökelek, T., Dal, H., Tercan, B., Aybirdi, Ö. & Necefoğlu, H. (2009*c*). *Acta Cryst.* E**65**, m1037–m1038. 10.1107/S1600536809027093 PMC2970058 21577401

[bb15] Hökelek, T., Dal, H., Tercan, B., Aybirdi, Ö. & Necefoğlu, H. (2009*d*). *Acta Cryst.* E**65**, m1365–m1366. 10.1107/S1600536809041208 PMC2970957 21578119

[bb16] Hökelek, T. & Necefoğlu, H. (1996). *Acta Cryst.* C**52**, 1128–1131.

[bb17] Hökelek, T., Yılmaz, F., Tercan, B., Gürgen, F. & Necefoğlu, H. (2009*a*). *Acta Cryst.* E**65**, m1416–m1417. 10.1107/S1600536809042640 PMC2971022 21578154

[bb18] Krishnamachari, K. A. V. R. (1974). *Am. J. Clin. Nutr.* **27**, 108–111. 10.1093/ajcn/27.2.108 4812927

[bb19] Macrae, C. F., Edgington, P. R., McCabe, P., Pidcock, E., Shields, G. P., Taylor, R., Towler, M. & van de Streek, J. (2006). *J. Appl. Cryst.* **39**, 453–457.

[bb20] Sheldrick, G. M. (2008). *Acta Cryst.* A**64**, 112–122. 10.1107/S0108767307043930 18156677

[bb21] Shnulin, A. N., Nadzhafov, G. N., Amiraslanov, I. R., Usubaliev, B. T. & Mamedov, Kh. S. (1981). *Koord. Khim.* **7**, 1409–1416.

[bb22] Spek, A. L. (2009). *Acta Cryst.* D**65**, 148–155. 10.1107/S090744490804362X PMC2631630 19171970

